# Single-Armed Longitudinal Intussusception Vasoepididymostomy in an Ex Vivo Rat Model With Video Demonstration

**DOI:** 10.7759/cureus.102332

**Published:** 2026-01-26

**Authors:** Yelena Akelina, Jan B Lombaard, Nicholas J Coleman, Joseph Abboud

**Affiliations:** 1 Simulation, Columbia University Vagelos College of Physicians and Surgeons, New York, USA; 2 Family Medicine, University of Calgary, Calgary, CAN

**Keywords:** intussusception vasoepididymostomy, microsurgical training, microsurgical vasoepididymostomy, rat model, single-armed microsuture, surgical education, vasectomy reversal, vasoepididymostomy

## Abstract

Microsurgical vasoepididymostomy (VE) is a technically demanding procedure that benefits from deliberate, reproducible skills training. Practice opportunities may be limited by the cost and availability of double-armed microsutures used in traditional two-needle intussusception techniques. Single-armed longitudinal intussusception vasoepididymostomy (SA-LIVE) reduces reliance on double-armed sutures. Although SA-LIVE has been described in prior publications, still images alone may not fully capture the sequence and microsurgical nuances; a video demonstration can improve clarity for learners. We present a step-by-step SA-LIVE technique for an ex vivo rat testis training model, using readily available single-armed 10-0 and 9-0 nylon sutures. The protocol standardizes the longitudinal intussusception sequence and is paired with a video demonstration illustrating each step to aid comprehension and reproducibility. This technical report is intended as a microsurgical training resource and does not include comparative outcome data.

## Introduction

Microsurgical vasoepididymostomy (VE) is a technically challenging procedure and requires deliberate skill development [1,2]. Practical barriers to training include the cost and limited availability of double-armed microsutures used in traditional two-needle techniques. The single-armed suture longitudinal intussusception vasoepididymostomy (SA-LIVE) modification has been described as an alternative to the traditional two-needle approach [2-5]. These modifications reduce the dependence on double-armed sutures.

Existing descriptions are often limited to still images and brief technique summaries. Video-based surgical education has been shown to improve skill acquisition [6,7]. In this education-focused technical report, we outline a step-by-step ex vivo rat testis training model for SA-LIVE using readily available single-armed sutures, supported by a video demonstration aligned to each step. This report is intended as a training resource for urologists and microsurgeons with established foundational microsurgical skills and does not report comparative outcomes.

## Technical report

The procedure and video were performed during the Advanced Microsurgery Course at the Microsurgery Training & Research Laboratory, NewYork-Presbyterian/Columbia University Irving Medical Center. Adult male laboratory rat testes (Sprague-Dawley male rat, four to five months old, ≈450 g; ex vivo) were collected post-euthanasia, from animals enrolled in protocols that received approval from Columbia University Medical Center Institutional Animal Care and Use Committee. No animals were euthanized specifically for the purposes of this technical report.

Specimens were used immediately after harvest or stored overnight (≤12 hours) submerged in 0.9% saline at 4°C.

For the procedure, the testis and epididymis were positioned on a pinboard. The free proximal end of the vas deferens was pinned to the board to reduce movement and better mimic in vivo handling. The setup is shown in the accompanying video.

Video 1 shows a single representative ex vivo procedure and has been edited for brevity.

**Video 1:** Single-armed longitudinal intussusception vasoepididymostomy (SA-LIVE) in an ex vivo rat testis training model

All procedures were performed under an operating microscope (Carl Zeiss OPMI MD on a S22 stand with a f=200 mm objective lens, 4x-24x magnification; Zeiss, Oberkochen, Germany).

Standard microsurgical instruments from Accurate Surgical & Scientific Instruments (ASSI; New York, USA) were used, including straight and curved micro forceps, microscissors, needle holders, and vessel dilators.

Sutures and adjuncts included: non-sterile microsurgery training nylon sutures on D-shaped 3/8-circle needles (10-0, DU-3 mm; 80 µm needle diameter; 15 cm suture length; product code NTDU00V; and 9-0, DU-4 mm; 120 µm needle diameter; 15 cm suture length; product code NTDY019; all sutures from Crownjun, Ichikawa, Japan), a sterile fine-tip skin marker (Medline, DYNJSM03H), methylene blue or indigo carmine dye, and non-sterile examination gloves.

Procedural steps

**Figure 1:**
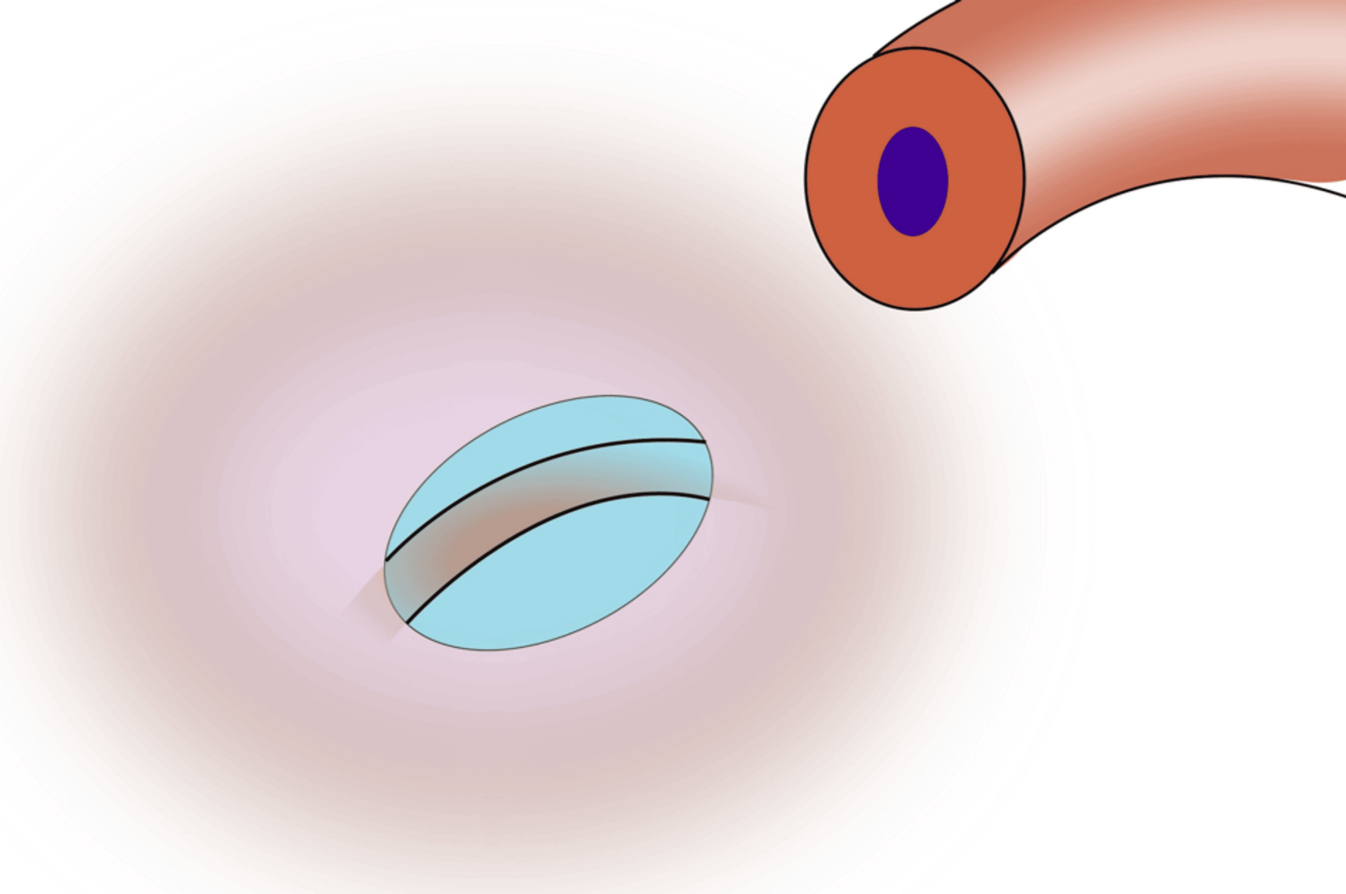
Create a window in the epididymal tunica with microscissors and blunt dissection Image credit: Original illustration created by Lombaard JB using Inkscape (v1.4, macOS/OSX; Inkscape Project, Massachusetts, United States).

**Figure 2:**
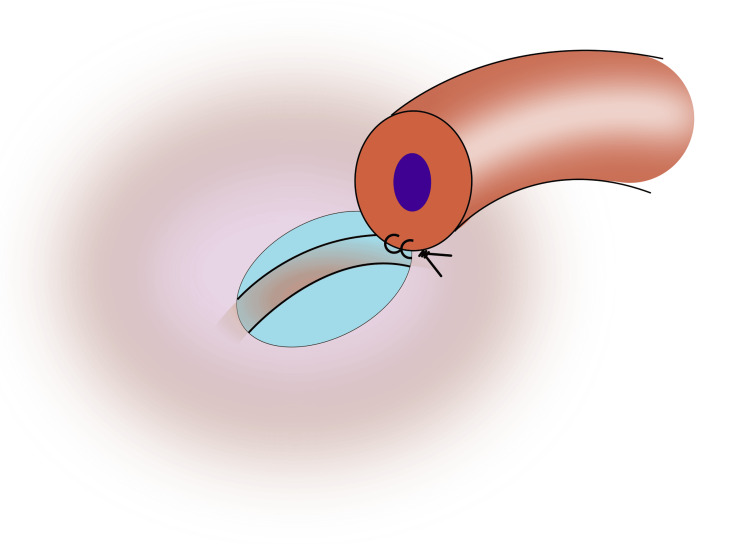
Anchor vas to the epididymal tunica with two to three interrupted 9-0 nylon sutures Image credit: Original illustration created by Lombaard JB using Inkscape (v1.4, macOS/OSX; Inkscape Project, Massachusetts, United States).

**Figure 3:**
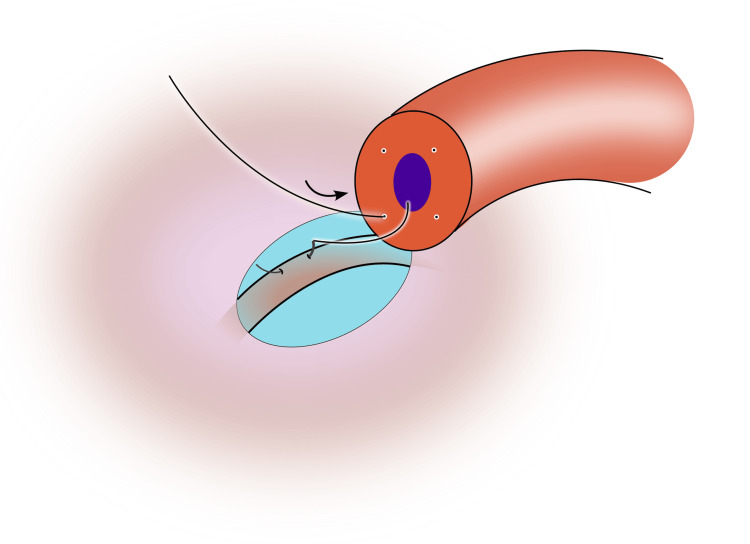
The first suture is passed through the lumen of the vas in an outside-in fashion, and the needle is parked in the epididymal tubule Image credit: Original illustration created by Lombaard JB using Inkscape (v1.4, macOS/OSX; Inkscape Project, Massachusetts, United States).

**Figure 4:**
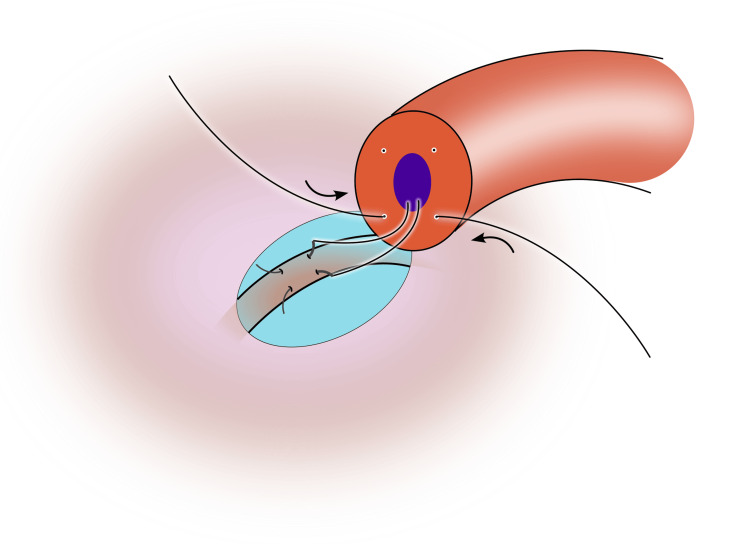
The second suture is placed on the contralateral side of the vas, and the needle is parked in the epididymal tubule, parallel to the first needle Image credit: Original illustration created by Lombaard JB using Inkscape (v1.4, macOS/OSX; Inkscape Project, Massachusetts, United States).

**Figure 5:**
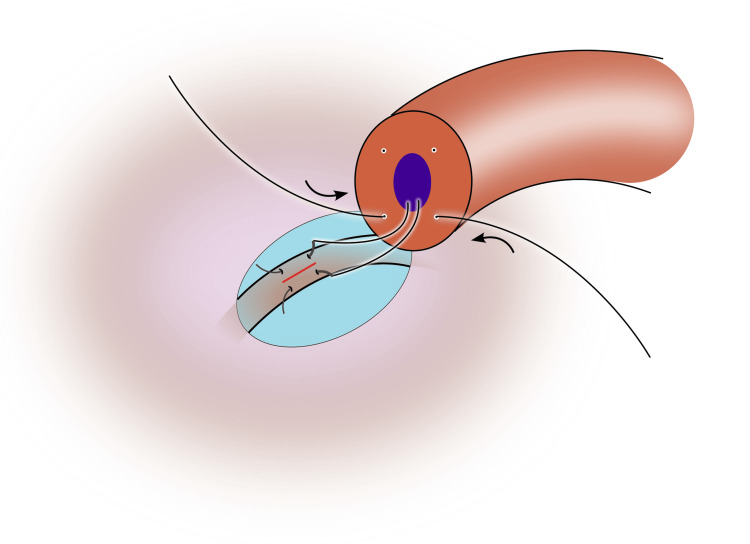
A tubulotomy is made between the needles with a microblade or microscissors Image credit: Original illustration created by Lombaard JB using Inkscape (v1.4, macOS/OSX; Inkscape Project, Massachusetts, United States).

**Figure 6:**
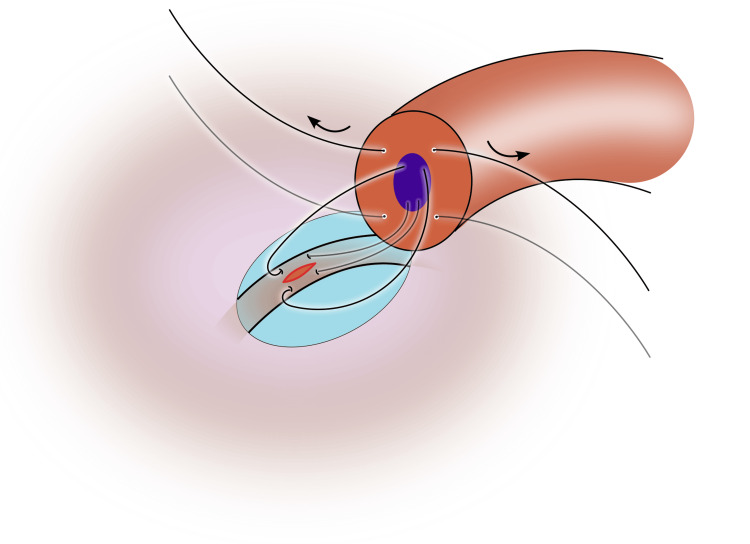
The sutures are passed back through the lumen of the vas, avoiding a cross-over Image credit: Original illustration created by Lombaard JB using Inkscape (v1.4, macOS/OSX; Inkscape Project, Massachusetts, United States).

**Figure 7:**
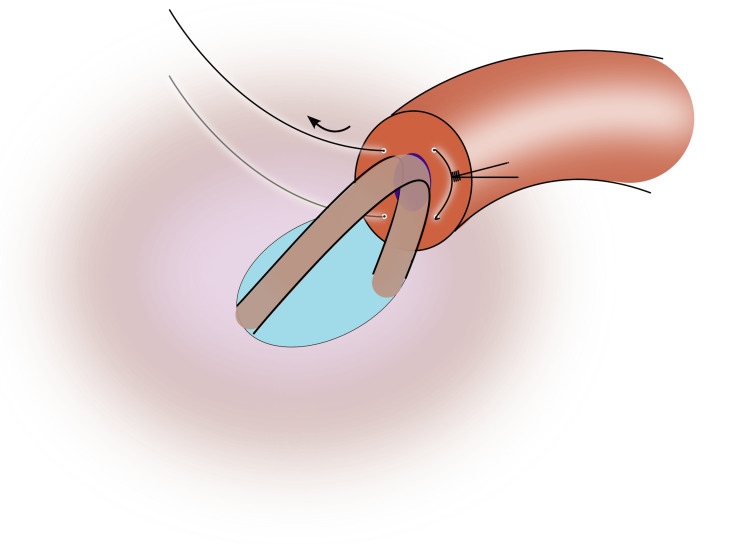
Gently pull on the sutures to intussuscept the epididymal tubule into the vasal lumen, then tie the first suture Image credit: Original illustration created by Lombaard JB using Inkscape (v1.4, macOS/OSX; Inkscape Project, Massachusetts, United States).

**Figure 8:**
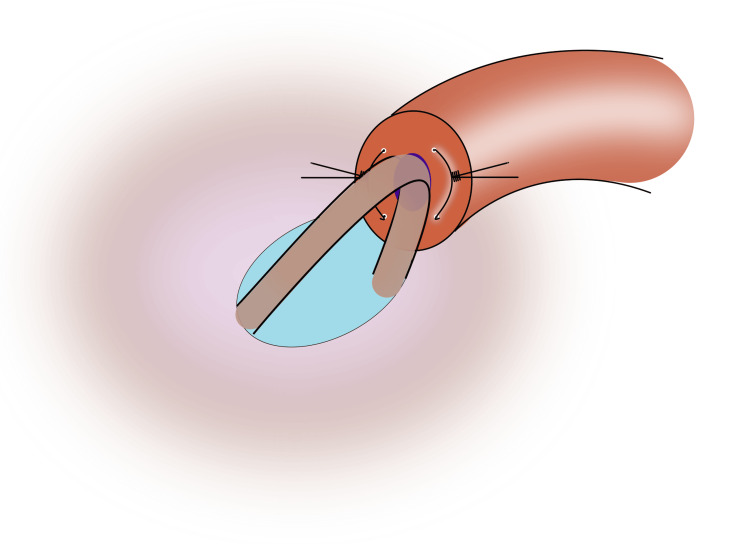
After tying the second suture, the epididymal tubule should be securely intussuscepted into the vasal lumen Image credit: Original illustration created by Lombaard JB using Inkscape (v1.4, macOS/OSX; Inkscape Project, Massachusetts, United States).

**Figure 9:**
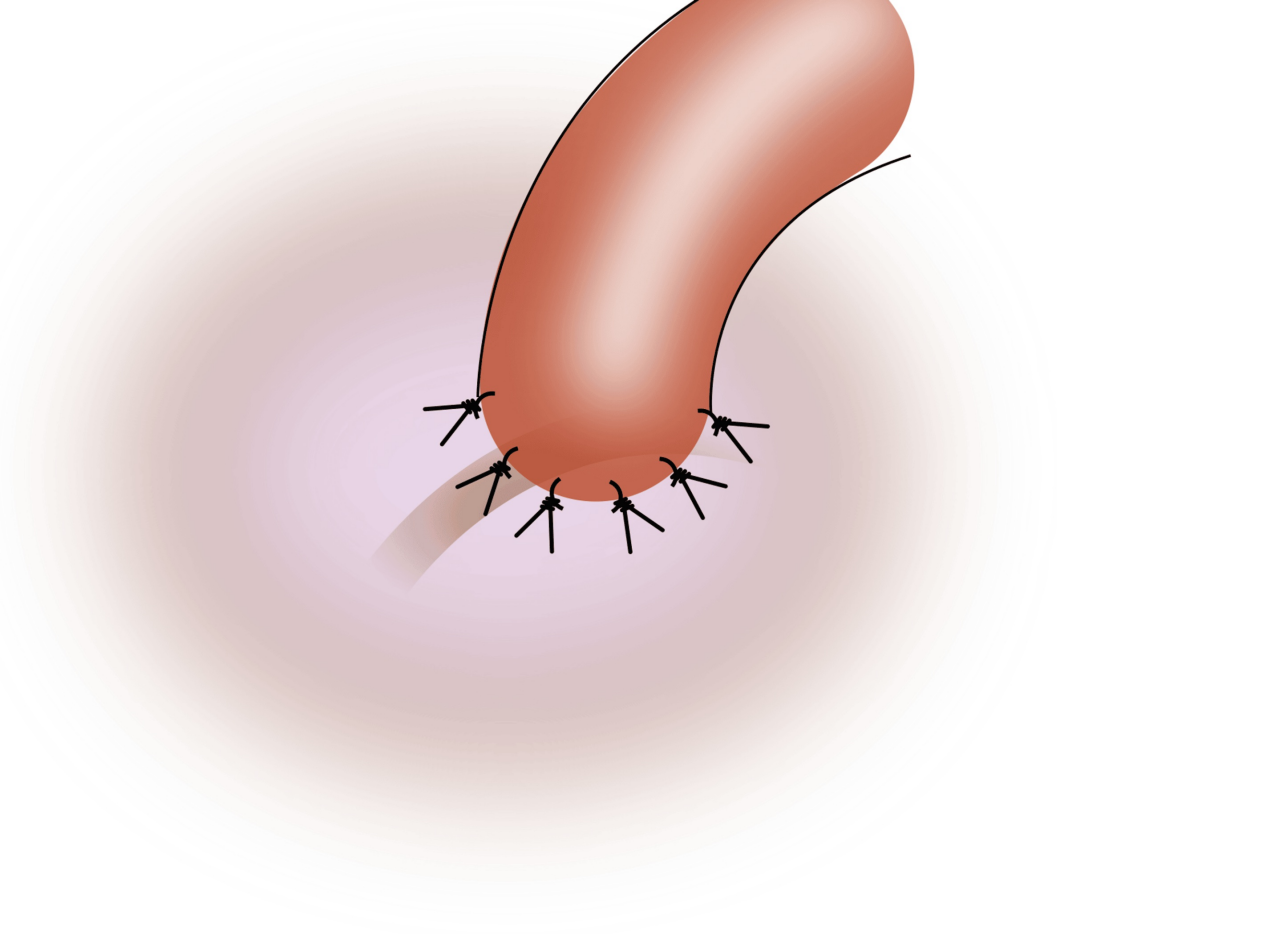
The vas is secured to the epididymal tunica with 9-0 nylon interrupted sutures to relieve tension from the anastomosis Image credit: Original illustration created by Lombaard JB using Inkscape (v1.4, macOS/OSX; Inkscape Project, Massachusetts, United States).

1) Gently dilate the vasal lumen with a vessel dilator and mark the lumen with a surgical marker; 2) Score the epididymal tunica with one tip of the microscissors, then use blunt dissection with the microscissors to create a window in the tunica (Figure 1);

3) Place a drop of methylene blue or indigo carmine dye in the window of the tunica to help identify the tubule and the edges of the epididymal tunica; 4) Anchor the posterior superficial muscularis of the vas to the epididymal tunica with two to three interrupted 9-0 nylon sutures (Figure 2);

5) Place the first 10-0 suture in a posterior quadrant. Pass the needle from the cut face through the vasal wall so it exits into the lumen, then advance longitudinally into the epididymal tubule and park the needle (Figure 3);

6) Place the second suture in the other posterior quadrant in a similar fashion as the first suture. Place the needle longitudinally into the epididymal tubule, parallel to the first, and leave the needles in place to prevent tubular collapse (Figure 4);

7) Make a longitudinal tubulotomy between the needles using a microblade or microscissors (Figure 5);

8) Advance the needles and pass them back through the vasal lumen in an inside-out fashion in the anterior quadrants (Figure 6);

Be careful to not cross the sutures; right posterior goes to right anterior, and left posterior goes to left anterior; 9) Tie the sutures to intussuscept the epididymal tubule (Figures 7, 8);

10) Secure the vas to the epididymal tunica with additional 9-0 nylon interrupted sutures (Figure 9) to complete the VE.

## Discussion

Using single-armed microsutures may lower the barriers to practicing VE by reducing dependence on double-armed sutures, which are typically single-use and can be cost-prohibitive for laboratory training [5]. This approach also allows for multiple practice anastomoses on an ex vivo rat specimen within a single session, improving cost-effectiveness for deliberate skills development.

An ex vivo rat model offers additional practical benefits for laboratory training. In our setting, testes were collected from animals used in other surgical procedures after euthanasia. They remained suitable for microsurgical handling even after being stored overnight in normal saline at around 4°C. This method allows for flexible scheduling and repeated practice without the logistical challenges of a live animal model.

We also adopted a posterior-to-anterior suture routing (e.g., posterior-to-anterior quadrants) as described by Zhao et al. [8,9], and Liu et al. [10]. This method avoids maneuvring the suture back beneath itself, as demonstrated by Monoski et al. [3], which may increase the risk of suture entanglement during setup. In our experience, the posterior-to-anterior routing was straightforward to perform and easy to reproduce. Our approach also avoids cutting and re-tying the suture to the tail, as demonstrated by Yuan et al. [4], thereby simplifying the overall process.

The ex vivo rat model has important model-specific limitations, including the absence of bleeding and subtle differences in tissue turgor compared to in vivo surgery. Nevertheless, it provides a useful platform for developing foundational microsurgical skills such as atraumatic tissue handling, needle control, suture management, and procedural sequencing.

A technique-specific limitation of the SA-LIVE technique in clinical practice is the preparatory work required before tubulotomy. If epididymal fluid at the selected site does not contain sperm, the anastomosis must be abandoned and a new site selected, increasing operative time. Despite this limitation, the microsurgical skills developed through SA-LIVE practice are transferable to traditional double-armed VE techniques.

Structured laboratory practice remains valuable, and the standardized steps outlined here, supported by a video demonstration, offer a reproducible framework for training and technique refinement.

## Conclusions

This technical note describes a reproducible, step-by-step method for SA-LIVE using an ex vivo rat testis model. By using readily available single-armed microsutures and a straightforward posterior-to-anterior suture route, the technique offers a practical and cost-effective platform for developing microsurgical skills. Although the model does not simulate in vivo bleeding or tissue turgor, it supports the acquisition of basic skills that are transferable to traditional double-armed VE techniques. The accompanying video demonstration aims to improve clarity, standardisation, and reproducibility for training.

## References

[1] Marks SH (2019). Vasectomy Reversal: Manual of Vasovasostomy and Vasoepididymostomy.

[2] Chan PT (2013). The evolution and refinement of vasoepididymostomy techniques. Asian J Androl.

[3] Monoski MA, Schiff J, Li PS, Chan PT, Goldstein M (2007). Innovative single-armed suture technique for microsurgical vasoepididymostomy. Urology.

[4] Yuan Y, Fang D, Lei H (2021). Rat model and validation of a modified single-armed suture technique for microsurgical vasoepididymostomy: Guo's SA-LIVE. Andrology.

[5] Li P, Ping P, Qian H (2014). AB149. A novel single-armed technique for microsurgical vasoepididymostomy: the reverse single-armed 2-suture longitudinal intussusception. Transl Androl Urol.

[6] Youssef SC, Aydin A, Canning A, Khan N, Ahmed K, Dasgupta P (2023). Learning surgical skills through video-based education: a systematic review. Surg Innov.

[7] Daniel R, McKechnie T, Kruse CC (2023). Video-based coaching for surgical residents: a systematic review and meta-analysis. Surg Endosc.

[8] Zhao L, Deng CH, Sun XZ (2013). A modified single-armed technique for microsurgical vasoepididymostomy. Asian J Androl.

[9] Zhao L, Tu XA, Zhuang JT, Chen Y, Wang WW, Zeng LY, Deng CH (2015). Retrospective analysis of early outcomes after a single-armed suture technique for microsurgical intussusception vasoepididymostomy. Andrology.

[10] Liu N, Li P, Zhi E (2020). A modified single-armed microsurgical vasoepididymostomy for epididymal obstructive azoospermia: intraoperative choice and postoperative consideration. BMC Urol.

